# Analysis of Blimp-1 and PD-1/PD-L1 Immune Checkpoint in an Autoimmune Thyroiditis Animal Model

**DOI:** 10.1155/2020/6543593

**Published:** 2020-02-17

**Authors:** Xue Zhang, Xiaoshu Lv, Mengya Chen, Haixia Liu

**Affiliations:** ^1^Department of Endocrinology and Metabolism, The Second Hospital of Dalian Medical University, Dalian, China; ^2^Department of Neurology, Affiliated Hospital of Qingdao University, Qingdao, China

## Abstract

**Objective:**

B lymphocyte-induced maturation protein 1 (Blimp-1) and programmed cell death protein 1 (PD-1) have opposing roles in the development of T cells; however, the mechanism of autoimmune thyroiditis- (AIT-) associated abortion is unclear. The present study investigated the expression of Blimp-1 and PD-1/PD-ligand 1 (PD-L1) in AIT-associated pregnancy loss and elucidated the related signaling pathway involving in the inflammatory response.

**Methods:**

An experimental fetal loss model with autoimmune thyroiditis was established after murine thyroglobulin- (mTg-) immunized CBA/J female mice mating with Balb/c males. ELISA was employed to investigate the TgAb level in the serum of CBA/J female mice. The expression of Blimp-1, PD-1/PD-L1, mammalian target protein rapamycin (mTOR), and Foxp3 proteins in the placenta and spleen was detected through immunofluorescence staining and western blotting.

**Results:**

ELISA indicated that the serum TgAb level in the mTg group was higher than that in the control group (*P* < 0.001). Fetal resorption rates increased in the mTg group compared with those in the control group (45.63% vs. 3.1%, *P* < 0.05). Blimp-1 levels in the placenta and spleen were higher in the AIT-related miscarriage group than in the control group. However, the expression of PD-1/PD-L1 and Foxp3 was significantly decreased in the placenta and spleen in the AIT-related miscarriage group.

**Conclusion:**

Blimp-1 participates in the pathogenesis of autoimmune thyroid disease-associated pregnancy loss through the inflammatory immune response, which is potentially mediated through the PD-1/PD-L1 signaling pathway.

## 1. Introduction

Autoimmune thyroiditis (AIT) is characterized by the presence of anti-thyroid antibodies, which include anti-thyroperoxidase (TPO-Ab) and anti-thyroglobulin antibodies (TG-Ab), as well as lymphocytes infiltrating the interior of the thyroid gland. AIT is the most prevalent autoimmune state that affects up to 5–20% of reproductive-age women [1]. Patients with anti-thyroid antibodies, even in the presence of euthyroidism, may be at a higher risk of adverse reproductive outcomes, including miscarriage and preterm birth [2–5]. The most common and severe adverse pregnancy outcome caused by AIT with euthyroidism is miscarriage; however, the mechanism has not been elucidated.

In 1990, Stagnaro-Green et al. [6] reported a 100% increase in the rate of miscarriage in unselected euthyroid women who were thyroid autoantibody positive in the first trimester of pregnancy. Subsequently, research has been conducted on this relationship. Haddow et al. [7] conducted a prospective study to analyze the relationship between thyroid autoantibodies in early pregnancy and adverse pregnancy outcomes in 10062 pregnant women, and the results revealed that pregnant women with elevated levels of thyroid autoantibodies in early pregnancy had a higher rate of preterm delivery (OR: 1.81; 95% CI: 1.17–3.68). In a meta-analysis, Chen and Hu [8] investigated the association between thyroid antibodies and miscarriage, and suggested that AIT was associated with an increased risk of spontaneous miscarriage in euthyroid women. Liu et al. [5] screened 3315 women in China at a low risk of thyroid dysfunction to evaluate the association of these conditions in early pregnancy with subsequent miscarriage and found that women with SCH and AIT are at an increased risk of miscarriage between 4 and 8 gestational weeks.

Regulatory T cells (Treg) play critical roles in maintaining self-tolerance and in preventing organ-specific autoimmunity, allergy, and allograft rejection [9, 10]. In pregnancy, they play key roles in fetal protection. The diminished number and suppressive capacity of Treg are highly correlated with abortion in mice [11, 12]. Programmed cell death protein 1 (PD-1) and PD-ligand 1 (PD-L1) are negative costimulators in T-cell activation [13, 14]. Francisco et al. [15] showed that PD-1/PD-L1 negative costimulatory signals can downregulate the expression of the PI3K-protein kinase B (Akt)-mammalian target protein rapamycin (mTOR) pathway, further regulating the differentiation, proliferation, metabolism, and survival of T cells. B lymphocyte-induced maturation protein 1 (Blimp-1), as a transcription factor, plays an inhibitory role in regulating the terminal differentiation of T cells [16]. Encoded by the prdm1 gene, Blimp-1 is well established as a master regulator of plasma cell differentiation and maintenance [17–19]. Moreover, Blimp-1 also plays a key role in spontaneous abortion induced by autoimmune diseases. Cimmino et al. [20] stated that the transcriptional repressor Blimp-1 was most highly expressed in Th2 cells, and that it directly repressed the transcription of three genes encoding proteins with critical roles in Th1 differentiation: IFN-*γ*, T-bet, and Bcl-6. Gong et al. [21] found that Blimp-1 expression was increased in the decidual membrane tissues of women with a history of recurrent miscarriage. It was speculated that the abnormal expression of Blimp-1 might lead to changes in inflammatory factors, which eventually lead to miscarriage.

For successful pregnancy, the maternal immune system must tolerate the semiallogeneic fetus. Immune tolerance is crucial for the growth and development of the fetus, and failure in immune tolerance may result in abnormal pregnancies. Quantitative and qualitative changes in the profile of T cells play an important role in the pathogenesis of infertility and increased pregnancy loss. However, few studies have investigated the interactions between Blimp-1 and PD-1/PD-L1 in abortion caused by AIT. In the present study, we evaluated the effect of Blimp-1 on the PD-1/PD-L1 pathway and in AIT-induced abortion.

## 2. Materials and Methods

### 2.1. Active Immunization with Thyroglobulin

All procedures were conducted in accordance with the National Institutes of Health Guide for the Care and Use of Laboratory Animals and were approved by the Animal Ethics Committee of Dalian Medical University. Sixty SPF female CBA/J mice (aged 4 weeks) were purchased from the experimental animal research institution at Peking Union Medical College, Chinese Academy of Medical Sciences (HuaFukang Biological Technology Co., Ltd., Marketing Department, Beijing, China). Murine thyroglobulin (mTg) was extracted from frozen mouse thyroids (KM mouse), as described by Imaizumi et al. [22]. To induce AIT, CBA/J mice were first immunized with mTg (75 *μ*g/mouse) in complete Freund's adjuvant at 5 weeks of age and were then challenged with mTg (75 *μ*g/mouse) in incomplete Freund's adjuvant at 7 weeks of age. After administering a booster dose of immunization for 4 weeks, CBA/J female mice were mated with Balb/c male mice; the presence of the female vaginal mucus plug was considered the 0.5th day of pregnancy. The mice bled and died on the 13.5th day of pregnancy.

### 2.2. Thyroid Function Tests

Total thyroxine (T4) and thyrotropin (TSH) levels were measured using a solid-phase chemiluminescence enzyme immunoassay (Immulite 1000, American DPC). The TSH and total T4 of control mice were measured individually. The functional sensitivity of the TT4 assay was 1 *μ*g/dL. The intra-assay coefficients of variation (CVs) of serum TSH and TT4 were 1.23%–3.38% and 1.26%–3.20%, respectively. Moreover, the interassay CV values were 1.57%–4.93% and 3.58%–6.67%, respectively.

### 2.3. Detection of mTg Antibody in Mouse Sera

Anti-Tg antibody was determined using ELISA (Elabscience Biotechnology Co., Ltd.). All samples were measured twice, and specific experimental steps were performed in accordance with the kit specifications.

### 2.4. Immunofluorescence Staining

Immunohistochemical staining of placental and spleen tissues for Blimp-1, PD-1, PD-L1, and mTOR was performed as follows: the tissues were embedded in paraffin following an established protocol [23] and were sliced into 5-*μ*m-thick sections. IHC for Blimp-1, PD-1, PD-L1, and FOXP3 was performed on tissue samples containing the placenta and spleen using our standard protocol. Negative controls were obtained by replacing the first antibody with 10% BSA in TBS. The antibodies used included anti-Blimp-1 polyclonal antibody (1 : 200, Arigo ARG 55270), anti-PD-L1 polyclonal antibody (1 : 400, Arigo ARG55930), anti-PD-1 polyclonal antibody (1 : 300, Biorbyt orb13641), anti-mTOR (1 : 200, Arigo ARG55930), and anti-FOXP3 monoclonal antibody (1 : 200, Biolegend RUO 126402).

### 2.5. Western Blot Analysis

Sample tissues were lysed with lysis buffer containing RIPA lysis buffer and PMSF (99 : 1). The supernatant was collected, and the protein concentration was measured using the BCA Protein Assay Reagent kit. A total of 20 *μ*g of protein was separated on 10% SDS-polyacrylamide gel and then transferred to polyvinylidene fluoride (PVDF) membranes (Immobilin-P, MilliporeCorp., MA, USA). After 2 hours of blocking with 10% nonfat milk at room temperature, the membranes were incubated overnight with the primary antibody of rabbit Blimp-1 (1 : 1000), PD-1 (1 : 500), PD-L1 (1 : 1000), mTOR (1 : 1000), or Foxp3 (1 : 1000). After washing, the membranes were incubated with anti-rabbit secondary antibodies conjugated to horseradish peroxidase (1 : 10000) for 2 hours at room temperature. Antigen-antibody complexes were detected using the enhanced chemiluminescence (ECL) reagent and were visualized on an imaging system (UVP Biospectrum 810, USA).

### 2.6. Statistical Analysis

The experimental data were analyzed using GraphPad Prism 5 software. Density values of protein bands obtained by western blot were detected using Image-pro Plus 6 software. All data were statistically analyzed using SPSS 19 or GraphPad Prism 5 software. Data were presented as mean ± SEM. *P* < 0.05 was considered statistically significant.

## 3. Results

### 3.1. Establishing a Fetal Loss Murine Model of Isolated Positive Maternal TgAb

**Figure 1:**
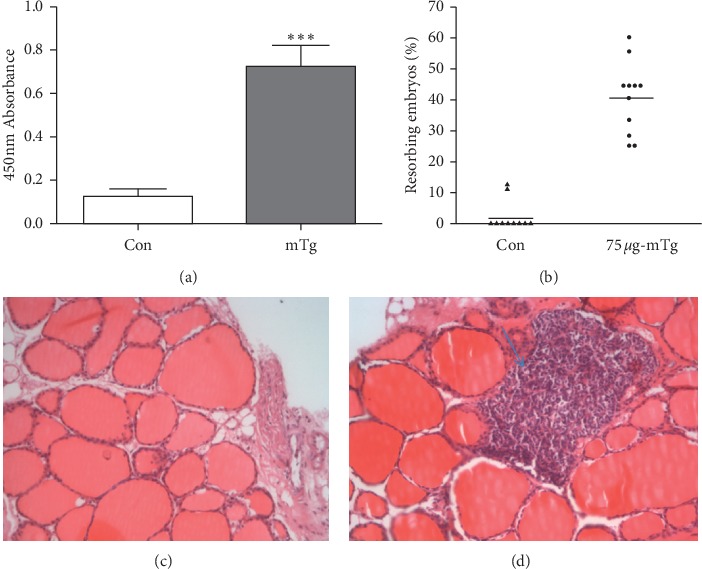
Comparison of serum TgAb levels between the two groups (a); comparison of embryo loss rate between the mTg group and Con group (b); normal thyroid tissue in the Con group (c); lymphocytic infiltration in the mTg group at 13.5 days of gestation (d).

No significant difference was found in TT4 and TSH levels between the mTg group and control group (*P*=0.078 and *P*=0.430, respectively, Table 1). The serum TgAb level in the mTg group was significantly higher than that in the control group (^*∗∗∗*^*P* < 0.001, Figure 1(a)). The volume of embryos in the mTg group was significantly smaller than that in the control group. Fetal resorption rates were increased in the mTg group compared with those in the control group (45.63% vs 3.1%, ^*∗*^*P* < 0.05) (Figure 1(b)). Histopathological examination of the thyroid showed that a large amount of lymphocytic infiltration occurred in the thyroid gland in the mTg group compared with that in the normal group. Moreover, the thyroid follicle was dilated and destroyed (Figures 1(c) and 1(d)). All of the aforementioned results indicated that the TgAb-positive fetal loss mouse model was successfully established.

### 3.2. Blimp-1 Expression in the Placenta and Spleen

**Figure 2:**
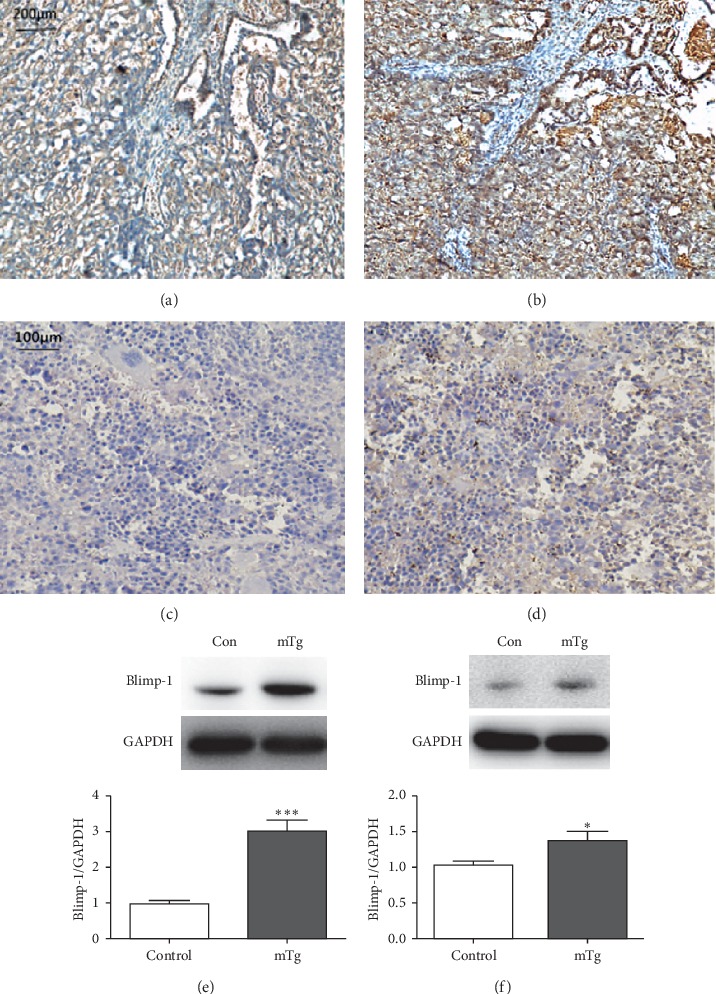
Blimp-1 expressed in each group, as revealed by immunohistochemistry: (a) lowly expressed in the control group in the placenta; (b) highly expressed in the mTg group in the placenta; (c) lowly expressed in the control group in the spleen; (d) highly expressed in the mTg group in spleen; western blot analysis and statistical results of protein quantification in the placenta (e) and spleen (f). Data are expressed as mean ± SEM (^*∗∗∗*^*P* < 0.001, ^*∗*^*P* < 0.05).

The expression of Blimp-1 in the placenta and spleen was evaluated in the mTg group and control group through immunohistochemistry and western blotting. The results of immunohistochemistry showed that Blimp-1 in the mTg group was significantly increased in the placenta, especially in the villus, which was the main part of the substance that the mother transported nutrients to the embryo and exchanged substances with the embryo (Figures 2(a) and 2(b)). Furthermore, western blotting revealed a significant increase in Blimp-1 in the mTg group compared with that in the control group (Figure 2(e); ^*∗∗∗*^*P* < 0.001). In accordance with the following results, we also found that the expression of Blimp-1 in the spleen was significantly increased in the mTg group compared with that in the control group (Figures 2(c), 2(d), and 2(f); ^*∗*^*P* < 0.05).

### 3.3. PD-1 and PD-L1 Expression in the Placenta and Spleen

**Figure 3:**
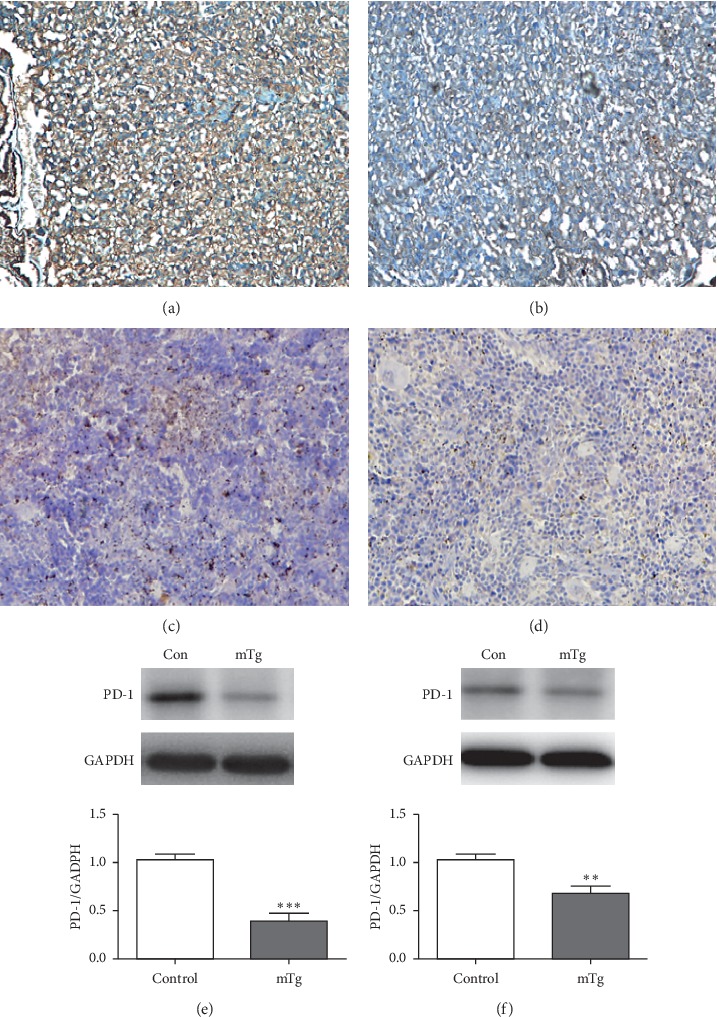
PD-1 expressed in each group, as revealed by immunohistochemistry: (a) highly expressed in the control group in the placenta; (b) lowly expressed in the mTg group in the placenta; (c) highly expressed in the control group in the spleen; (d) lowly expressed in the mTg group in the spleen; western blot analysis and statistical results of protein quantification in the placenta (e) and spleen (f). Data are expressed as mean ± SEM (^*∗∗∗*^*P* < 0.001, ^*∗∗*^*P* < 0.01).

**Figure 4:**
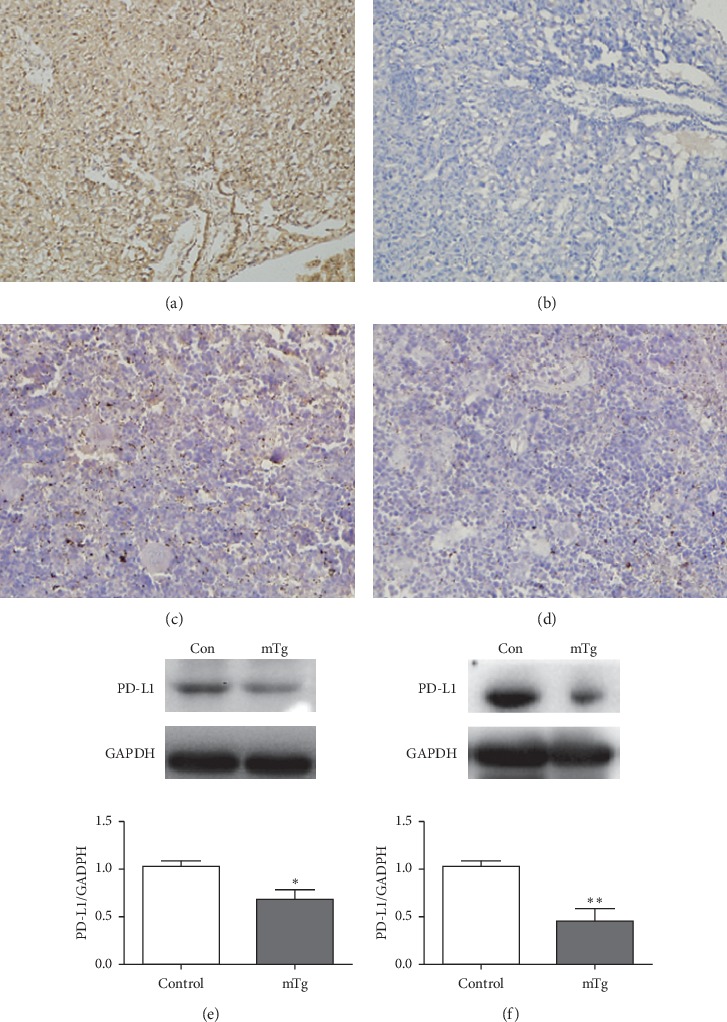
PD-L1 expressed in each group, as revealed by immunohistochemistry: (a) highly expressed in the control group in the placenta; (b) lowly expressed in the mTg group in the placenta; (c) highly expressed in the control group in the spleen; (d) lowly expressed in the mTg group in spleen; western blot analysis and statistical results of protein quantification in the placenta (e) and spleen (f). Data are expressed as mean ± SEM (^*∗∗*^*P* < 0.01, ^*∗*^*P* < 0.05).

We determined the expression of PD-1/PD-L1 in the placenta and spleen through immunohistochemistry and western blotting. The results of immunohistochemistry and western blotting revealed that the expression of PD-1 and PD-L1 in the placenta was decreased in the mTg group compared with the control group (^*∗∗∗*^*P* < 0.001 and ^*∗*^*P* < 0.05, respectively) (Figures 3(a), 3(b), and 3(e); Figures 4(a), 4(b), and 4(e)). The expression of PD-1 and PD-L1 in the spleen was consistent with that in the placenta (^*∗∗*^*P* < 0.01) (Figures 3(c), 3(d), and 3(f); Figures 4(c), 4(d), and 4(f)).

### 3.4. mTOR Expression in the Placenta and Spleen

**Figure 5:**
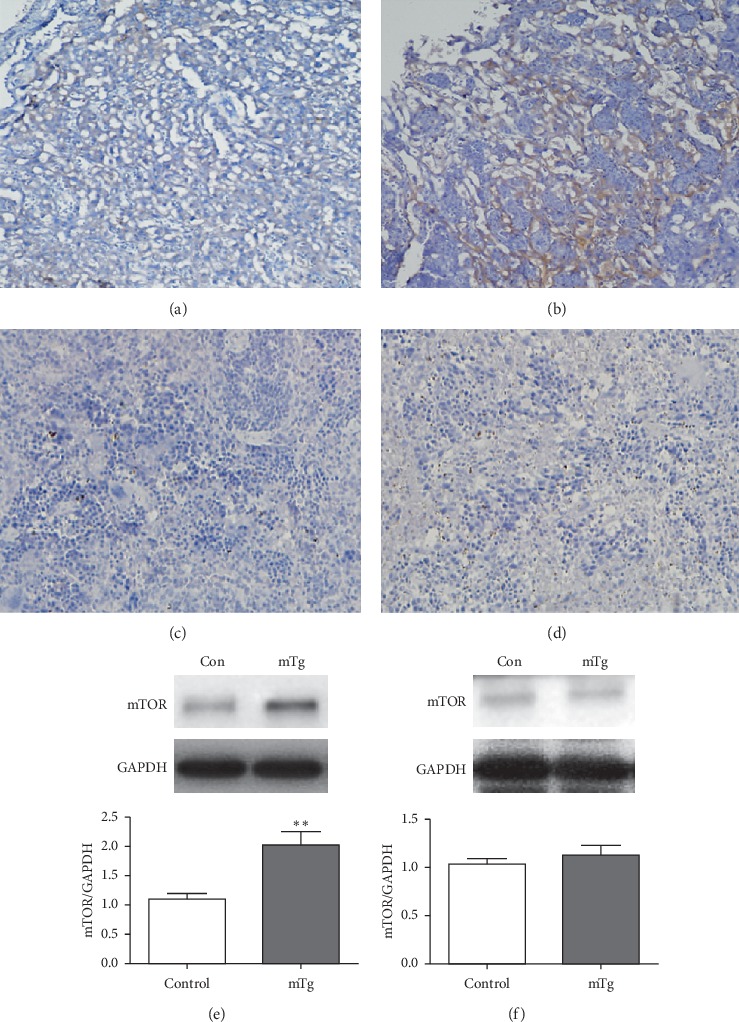
mTOR expressed in each group, as revealed by immunohistochemistry: (a) lowly expressed in the control group in the placenta; (b) highly expressed in the mTg group in the placenta; (c, d) there was no significant difference in mTOR between the control group and mTg group in the spleen; western blot analysis and statistical results of protein quantification in the placenta (e) and spleen (f). Data are expressed as mean ± SEM (^*∗∗*^*P* < 0.01).

Immunohistochemistry demonstrated that the mTOR level in the placenta increased in the mTg group compared with the control group (Figures 5(a) and 5(b)). Western blotting also revealed an increase in mTOR in the mTg group compared with the control group (^*∗∗*^*P* < 0.01; Figure 5(e)). However, the expression of mTOR in the spleen detected by western blotting was slightly different, and no difference was observed between the mTg group and control group (*P* > 0.05; Figure 5(c), 5(d), and 5(f)).

### 3.5. Treg Expression in the Placenta and Spleen

**Figure 6:**
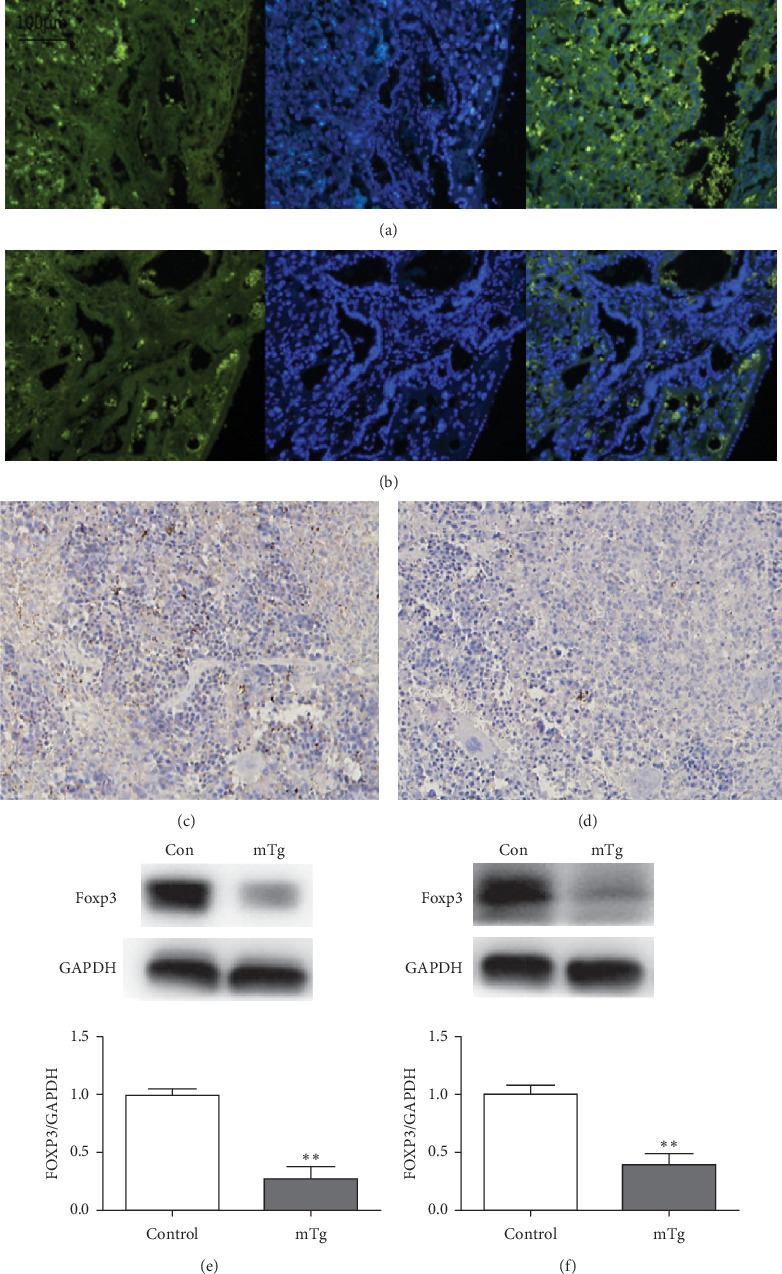
Foxp3 expressed in each group: (a) highly expressed in control group in the placenta, as revealed by immunofluorescence; (b) lowly expressed in the mTg group in the placenta, as revealed by immunofluorescence; (c) highly expressed in the control group in the spleen, as revealed by immunohistochemistry; (d) lowly expressed in the mTg group in the spleen, as revealed by immunohistochemistry; western blot analysis and statistical results of protein quantification in the placenta (e) and spleen (f). Data are expressed as mean ± SEM (^*∗∗*^*P* < 0.01).

Foxp3 is a molecular marker of Treg and detecting the Foxp3 level can reveal the Treg status. In the present study, we found that the expression of Foxp3 in the placenta in the mTg group was lower than that in the control group (^*∗∗*^*P* < 0.01; Figure 6(a), 6(b), and 6(e)). Moreover, the expression of Foxp3 in the spleen in the mTg group was significantly lower than that in the control group (^*∗∗*^*P* < 0.01; Figure 6(c), 6(d), and 6(f)).

## 4. Discussion

The relationship between AIT and abortion is an important research topic. Several hypotheses have been proposed for the pathogenesis of increased pregnancy loss in women with AIT and include different mechanisms that can be classified as thyroid dependent and thyroid independent [24, 25]. In this study, we successfully established a TgAb-positive abortion mouse model. The results confirmed lymphocyte infiltration, high TgAb concentration, and abnormal embryo absorption in the experimental group.

In the successfully established simple thyroid autoantibody-positive pregnant mice, our study showed that the expression of PD-1, PD-L1, and Foxp3 in the placenta was reduced, whereas the expression of Blimp-1 was increased, and the proportion was unbalanced. Similarly, in the spleen, PD-1, PD-L1, and Treg levels decreased, while the Blimp-1 level increased. Moreover, no statistical difference was observed in mTOR in the spleen between the mTg and control groups. All of these proteins are involved in the maintenance of pregnancy. Abnormal changes in immune checkpoints may result in an imbalance of immune tolerance at multiple levels, which may lead to abortion, and the mTOR energy metabolism signaling pathway may only play a role in the maternal-fetal interface.

At present, there are few studies on the main role of Blimp-1 in adverse pregnancy outcomes. Blimp-1, which has a molecular weight of 98 kDa and is encoded by the PRDM1 gene, is a transcriptional repressor that is required for the terminal differentiation of B cells into plasma cells. It also has a role in regulating T cells, including the attenuation of T-helper type 1 (Th1) cells [26–28] and downregulation of IL-2 in CD4+ cells and affecting the development and suppressive function of Treg. Although accumulating data indicate the critical roles of Blimp-1 in T cell function [29–31], the pathological mechanism of Blimp-1 in AIT remains unclear. Gong et al. [21] found that Blimp-1 expression was increased in the decidual membrane tissues of women with a history of recurrent miscarriage. It was speculated that the abnormal expression of Blimp-1 might lead to changes in inflammatory factors, which eventually lead to miscarriage. Savitsky et al. [18] showed that Blimp-1 repressed PD-1 through a feed-forward repressive circuit by regulating PD-1 directly and by repressing the expression of NFATc1, an activator of PD-1 expression. Blimp-1 binding induced a repressive chromatin structure at the PD-1 locus, which may lead to the removal of NFATc1 from its site. In our study, we infer that Blimp-1 regulates T cells through the PD-1/PD-L1 pathway at the peripheral and maternal-fetal interfaces. Furthermore, mTOR acts as an energy metabolism pathway connecting the PD-1/PD-L1 immune checkpoint with T cells.

PD-1/PD-L1 plays a crucial role in the occurrence and development of immune tolerance [32–34]. In the present study, the results showed that the expression of PD-1 and PD-L1 in the spleen and placenta was decreased in mTg-positive mice. PD-1/PD-L1 regulates immune tolerance at the maternal-fetal and peripheral interfaces to maintain the pregnancy status. When the PD-1/PD-L1 pathway is abnormal, the imbalance of immune regulation leads to abortion. Guleria et al. [35] showed that the excessive immune response of T cells would have adverse effects on the fetus, and PD-L1 could inhibit this reaction and maintain a healthy pregnancy state. Recent data in vitro demonstrated that coculturing PD-L1-Ig beads with naive CD4+ T cells in the presence of anti-CD3 and TGF-*β* induced the conversion of naive CD4+ T cells into CD4+Foxp3+ Tregs; in addition, PD-L1-Ig also enhanced the expression of Foxp3.

In previous studies, the experimental results showed that the negative regulation function of PD-1/PD-L1 on maternal-fetal tolerance is achieved by regulating Treg [36–38]. The effect of PD-1/PD-L1 on Treg is mainly achieved by inhibiting the signaling of protein kinase B/mammalian target protein rapamycin (AKT/mTOR). mTOR is an important signaling pathway of cellular energy metabolism. Francisco et al. [15] showed that the development of PD-L1 Treg is mediated by the downregulation of the key signaling molecules phospho-Akt, mTOR, and S6. In melanoma, PD-1 may negatively regulate melanoma cells by inhibiting downstream mTOR signaling effector molecules, indicating the crucial role of PD-1/PD-L1 in the mTOR signaling pathway [39]. Significantly, PD-L1 can promote the differentiation of Treg and maintain the function of induced Treg by sustaining and enhancing Foxp3 expression in iTreg. PD-L1 induces iTreg by inhibiting the Akt/mTOR signaling cascade, thereby flipping the “molecular switch” in a naive CD4+ T cell toward Treg development. The novel role of PD-L1 in the maintenance and induction of iTreg is that PD-L1 acts as a promising therapeutic target for controlling Treg plasticity.

## 5. Conclusion

To summarize, our results suggest that increased pregnancy loss in autoimmune thyroiditis is associated with upregulation of Blimp-1 and downregulation of PD-1/PD-L1 signaling pathway. Blimp-1, PD-1/PD-L1, mTOR, and Treg play regulatory roles in maintaining normal pregnancy, protecting the mother and embryo and regulating immune tolerance. The increase in Blimp-1 expression in mice with AIT may inhibit the PD-1/PD-L1 pathway and then inhibit the mTOR signal, further affecting the function and number of Treg. Maternal rejection of embryos leads to adverse pregnancy outcomes. These results provide new possibilities and targets for the treatment of miscarriage caused by AIT.

## Figures and Tables

**Table 1:** The comparison of the serum levels of TSH and TT4 ($\bar{x}\pm \text{SEM}$).

Groups	TSH (mIU/L)	TT4 (*μ*g/dL)
mTg group (*n* = 15)	0.31 ± 0.03	4.70 ± 0.91
Con group (*n* = 15)	0.33 ± 0.05	5.11 ± 0.25
*P* value	0.430	0.078

Data are expressed as mean ± SEM. TT4, total thyroxine; TSH, thyroid stimulating hormone; *P* < 0.05 was considered statistically significant.

## Data Availability

The data used to support the findings of this study are available from the corresponding author upon request.
